# Global gray matter changes in posterior cortical atrophy: A serial imaging study

**DOI:** 10.1016/j.jalz.2011.09.225

**Published:** 2012-11

**Authors:** Manja Lehmann, Josephine Barnes, Gerard R. Ridgway, Natalie S. Ryan, Elizabeth K. Warrington, Sebastian J. Crutch, Nick C. Fox

**Affiliations:** Dementia Research Centre, UCL Institute of Neurology, University College London, London, United Kingdom

**Keywords:** Posterior cortical atrophy, Magnetic resonance imaging, Longitudinal, Boundary shift integral, Voxel-based morphometry, Classification

## Abstract

**Background:**

Posterior cortical atrophy (PCA) is a neurodegenerative condition predominantly associated with Alzheimer’s disease (AD) pathology. Cross-sectional imaging studies have shown different atrophy patterns in PCA patients compared with typical amnestic Alzheimer’s disease (tAD) patients, with greatest atrophy commonly found in posterior regions in the PCA group, whereas in the tAD group, atrophy is most prominent in medial temporal lobe regions. However, differential longitudinal atrophy patterns are not well understood.

**Methods:**

This study assessed longitudinal changes in brain and gray matter volumes in 17 PCA patients, 16 tAD patients, and 18 healthy control subjects. Both patient groups had symptom durations of approximately 5 years.

**Results:**

Progressive gray matter losses in both PCA and tAD patients were relatively widespread throughout the cortex, compared with control subjects, and were not confined to areas related to initial symptomatology. A multivariate classification analysis revealed a statistically significant group separation between PCA and tAD patients, with 72.7% accuracy (*P* < .01).

**Conclusion:**

Progression from an initially focal presentation to a more global pattern suggests that these different clinical presentations of AD might converge pathologically over time.

## 1 Introduction

Alzheimer’s disease (AD) is the most common cause of dementia in people aged >65 years [1], and it is estimated to affect 81 million people worldwide by 2040 [2]. It is typically characterized by an insidious onset of memory impairment that progresses to involve multiple cognitive domains [3]. This amnestic presentation is in accordance with histopathological and structural imaging studies showing early medial temporal lobe involvement. Intracellular neurofibrillary tangles (a hallmark of AD) are first seen in the transentorhinal cortex and then in the hippocampus [4]. Neuroimaging evidence suggests atrophy of medial temporal lobe structures is an early feature of typical amnestic Alzheimer’s disease (tAD) and predicts progression to AD in individuals with mild cognitive impairment (MCI) [5,6].

However, less typical forms of AD have been described in which memory is not the primary deficit [7]. In particular, posterior presentations of AD are increasingly recognized. Individuals may present with visuospatial, visuoperceptual, praxis, calculation, and spelling difficulties, which implicate early parietal and occipital lobe involvement [8,9]. The posterior pattern of atrophy with which these presentations are associated has led to the term posterior cortical atrophy (PCA). The underlying pathology in these patients is most commonly AD, which is found concentrated in the occipital and parietal lobes [10,11]. A smaller proportion of PCA cases may be underpinned by other conditions such as dementia with Lewy bodies, corticobasal degeneration, or, rarely, Creutzfeldt–Jakob disease [12,13].

Cross-sectional magnetic resonance imaging (MRI) studies have shown that PCA patients have greater parietal atrophy and less medial temporal and hippocampal atrophy compared with tAD patients [14,15]. Longitudinal studies investigating how this atrophy pattern changes over time are lacking. In particular, longitudinal investigations are needed to assess whether the focal patterns of atrophy remain confined to these regions or whether they become more global. The areas of greatest change may not be the areas that are most atrophied initially. Results from longitudinal studies of PCA may allow for a better understanding of the natural history of both PCA and AD.

A number of techniques have been developed to study longitudinal changes in specific structures and across the whole brain. A widely used method to quantify changes over time in whole brain volume is the boundary shift integral (BSI; [16]). This method has been applied to measure whole brain atrophy in a range of neurological disorders, including AD [17], frontotemporal lobar degeneration [18], Huntington disease [19], and progressive supranuclear palsy [20]. This measure is also used as an outcome measure in clinical trials of therapies targeted at AD pathology [21,22]. Another method that assesses local volumetric changes over time across the whole brain uses nonlinear (fluid) registration and estimates volume changes from the deformation fields needed to match an individual’s serial scans [23]. This method can be used in combination with voxel-based morphometry (VBM) to assess local changes in gray matter, white matter, and cerebrospinal fluid (CSF), and has previously been applied to AD [24,25] and frontotemporal lobar degeneration [26].

The aim of the current study was to assess longitudinal changes in whole brain volume and gray matter volume in PCA, and compare these with structural changes over time in groups of tAD patients and healthy control subjects. Specifically, two possible outcomes were predicted: patterns of atrophy progression are either *(i)* focal, with PCA patients showing greater atrophy changes in posterior regions compared with control subjects and tAD patients, and tAD showing greater atrophy changes in medial temporal lobe regions compared with control subjects and PCA patients, leading to significantly different patterns of atrophy changes between PCA and tAD patients, or *(ii)* global, with PCA and tAD patients showing widespread changes of atrophy compared with control subjects, and only subtle differences in the direct comparison of PCA and tAD patients. Understanding the progression of atrophy in PCA patients may improve diagnosis and the quality of prognostic information given to these patients.

## 2 Materials and methods

### 2.1 Subjects

The study included 17 PCA patients, 16 tAD patients, and 18 healthy control subjects. All subjects were identified retrospectively from a clinical database. Demographics of the subjects are summarized in Table 1. All clinically affected subjects had attended the Specialist Cognitive Disorders Clinic at the National Hospital for Neurology and Neurosurgery, London, United Kingdom. Informed consent was obtained from all subjects, and the study had local ethics committee approval. All PCA and tAD patients (100%) and 12 control subjects (67%) had been included in a previous cross-sectional imaging study [15]. All subjects underwent comprehensive clinical and neuropsychological assessment. The clinical notes of all PCA patients were reviewed by a clinician to identify initial symptoms and clinical findings (Table 2). An overview of the proportion of PCA and tAD patients showing impaired performance (below the fifth percentile) on neuropsychological tests of memory, arithmetic, naming, spelling, speed (letter cancellation), and visuospatial and visuoperceptual processing is provided in Table 3.

#### 2.1.1 Inclusion criteria

All patients had to have at least two MRI scans, approximately 1 year apart, on the same scanner to be included in the study. Individuals were only included in the PCA group if clinical assessment and investigation provided no indication of a non-AD dementia (e.g., dementia with Lewy bodies, corticobasal degeneration). All PCA patients fulfilled the clinical criteria proposed by Mendez et al and Tang-Wai et al [34,35]. In addition, patients had to fulfill a set of behavioral criteria at some stage in their clinical history, chosen to assess quantitatively the cognitive domains most commonly affected in PCA patients. Patients had to demonstrate deficits on at least two of four standardized tests of parietal/occipitoparietal dysfunction. The Number Location and Object Decision subtests from the Visual Object and Space Perception battery were used to detect visuospatial and visuoperceptual dysfunction, respectively, skills commonly impaired after right or bilateral parietal damage [29]. The Graded Difficulty Arithmetic and the Graded Difficulty Spelling tests assess numeracy and literacy functions, which are commonly compromised by left parietal damage [31,33]. This decision of failing at least two of the aforementioned posterior cortical function tests was made to ensure that criteria are sensitive to parietal dysfunction, while not being so specific as to exclude PCA patients with either a relatively asymmetric or focal syndrome at onset. In addition, patients had to demonstrate relatively preserved episodic memory (below the fifth percentile on a short Recognition Memory Test; [27]), to distinguish patients with PCA from those with a more typical amnestic presentation of AD.

All tAD patients fulfilled National Institute of Neurological and Communicative Disorders and Stroke and the Alzheimer's Disease and Related Disorders Association criteria for probable AD, with recently proposed revisions [3,36], and additionally demonstrated significant episodic memory impairment (below the fifth percentile on verbal and visual Recognition Memory Tests; [37]).

### 2.2 MRI acquisition

All scans were acquired on the same scanner, and scan acquisition was consistent between time points for each subject. T1-weighted volumetric MR scans were obtained on a 1.5-T General Electric (Milwaukee, WI) Signa scanner using an inversion recovery-prepared fast spoiled gradient recalled sequence, with a 24-cm field of view and 256 × 256 matrix providing 124 contiguous 1.5-mm-thick slices in the coronal plane. Scanning parameters were as follows: repetition time = 12 ms or 15 ms, echo time = 5.4 ms, and inversion time = 650 ms.

### 2.3 Registration of repeat to baseline image

Both baseline and repeat brain regions were delineated using a semiautomated technique based on intensity thresholding and mathematical morphological operations [38]. The delineated repeat brain images were then registered to baseline images using 12-degree-of-freedom registration [39,40]. Differential bias correction (DBC) was applied to the registered baseline and repeat images using a kernel radius of 5 voxels, to correct for differences in intensity inhomogeneity artifacts between the two images [41].

### 2.4 Boundary shift integral

The registered and DBC-corrected scan pairs were used to calculate volume changes using the BSI algorithm, as described in Leung et al (KN-BSI; [42]).

### 2.5 Fluid registration

The registered and DBC-corrected scan pairs were cropped using subject-specific masks to exclude nonbrain regions (e.g., neck and eye), while including lateral ventricular CSF, gray matter, white matter, and a layer of brain surface CSF. The fluid registration [23] warps each individual’s repeat image to match the corresponding baseline image based on a physical model of a compressible viscous fluid. Fluid registrations were run until a stopping criterion based on the derivative of the cost function of the registration was reached [43]. The fluid registration generates a detailed deformation field for each subject. The amount of voxel-level expansion or contraction was extracted from each deformation field by computing the Jacobian determinant at each voxel (i.e., the determinant of the gradient of the deformation field). The Jacobian determinants were then log-transformed, and the resulting “voxel-compression map” for each subject was stored.

### 2.6 Voxel-based morphometry

The voxel-compression maps as well as the baseline images were converted to Neuroimaging Informatics Technology Initiative format (http://nifti.nimh.nih.gov) to process them using SPM8 (Statistical Parametric Mapping [Wellcome Trust Centre for Neuroimaging, UCL, London, UK], version 8, http://www.fil.ion.ucl.ac.uk/spm), executed in MATLAB 7.2 (MathWorks, Sherborn, MA). The baseline images were segmented into gray matter, white matter, and CSF using SPM8’s new segment toolbox with default settings. Segmentations were produced with rigid alignment to standard space (the international consortium for brain mapping template) and resampled to 1.5-mm isotropic voxels for use with Diffeomorphic Anatomical Registration Through Exponentiated Lie algebra (DARTEL) [44]. DARTEL then iteratively registered the gray matter segments to an evolving estimate of their groupwise average [45]. The transformation parameters required to warp the original segments to the final template (i.e., rigid alignment plus DARTEL warping) were applied to the gray matter segments and the voxel-compression map images to normalize them to the groupwise average template. The warped gray matter segments were then thresholded at a value of 0.5 to produce binary masks that were then used to multiply the voxel-compression map images to generate separate gray matter voxel-compression maps for each subject. These gray matter voxel-compression maps were smoothed using an isotropic Gaussian kernel of 6-mm full width at half maximum to render the data more normally distributed and to correct for minor misalignments after normalization.

### 2.7 Statistical analysis

Differences in measures of atrophy between groups were analyzed using a general linear model (GLM) with a three-level group factor, adjusting for covariates, as specified in later text.

#### 2.7.1 Boundary shift integral

BSI measures were annualized and differences in rates of atrophy between groups were analyzed using a GLM with age and sex included as covariates.

#### 2.7.2 Voxel-based morphometry

Longitudinal volume changes were analyzed using a GLM, adjusting for age, sex, and interval between time points. An explicit mask was applied to include only voxels for which the intensity was at least 0.1 in at least 80% of the images [46]. Statistical significance of the group differences was tested using a multiple comparison correction to control the false discovery rate (FDR; [47]) at *P* = .05. Results are displayed as overlays on a study-specific template, which was created by normalizing all original images using the DARTEL transformations and calculating the average of the warped brain images. Group differences are presented either as statistical difference maps (FDR-corrected) or as effect size maps (using Pearson correlation coefficients).

### 2.8 Support vector machine

A linear support vector machine (SVM; [48]) was used to assess how well patterns of gray matter volume derived from the VBM analysis can separate the three groups (control subjects, PCA patients, and tAD patients). The SVM analysis was implemented with LIBSVM version 2.89 [49] under MATLAB (version 7.2.0; MathWorks). SVMs identify an optimal separating hyperplane in this space such that subjects from each group lie as far as possible from the hyperplane, on opposite sides. After the hyperplane has been defined, scores can be generated by projecting the points onto the normal of the hyperplane; the direction of the normal can be visualized as an image, showing the relative weights and signs of vertices’ contributions to the classifier scores. We used the C-SVM formulation, which uses a two-level nested cross-validation to optimize the misclassification penalty parameter C using a leave-one-out procedure within the main leave-one-out loop [50]. This ensures an unbiased estimation of generalization accuracy by leaving each scan in turn entirely out of the training procedure.

## 3 Results

### 3.1 Subjects

As shown in Table 1, there was no evidence of a difference in sex distribution or age between the PCA and control groups (*P* > .8, both tests). However, the tAD group was older compared with control and PCA groups (both *P* < .001). As expected, there was a significant difference in baseline and follow-up Mini-Mental State Examination (MMSE) between PCA and control groups (*P* < .0001), and between tAD and control groups (*P* < .0001); however, no significant difference was found between PCA and tAD groups (*P* = .2 at baseline, *P* = .3 at follow-up). Both PCA and tAD patients showed a similar decline in MMSE from baseline to follow-up (2.6 points in PCA, 2.4 points in tAD, with similar, ∼1 year, intervals between time points). Furthermore, no significant differences were found for total intracranial volume (TIV) (*P* = .8 across groups, *P* > .5 between groups). Whole brain volume at baseline and follow-up, adjusted for TIV, was significantly different across groups (*P* < .0001 for both baseline and follow-up) and between each patient group and the control group (*P* < .001 for both PCA and tAD patients). No significant differences in baseline and follow-up whole brain volume were found between PCA and tAD patients (*P* = .7 at baseline, *P* = .8 at follow-up).

The most commonly reported initial symptoms (Table 2) in the PCA group were difficulties with arithmetic (71%), spelling (41%), and locating objects (41%). A high proportion of PCA patients also reported difficulties with “memory” (47%), whereas a much lower proportion showed impaired performance on memory tasks on formal neuropsychological testing at visit 1 (29%; Table 3). The most commonly reported clinical feature on neurological examination in the PCA group was dyspraxia (47%), followed by visual disorientation (24%). Neuropsychological testing (Table 3) revealed that all PCA patients showed impaired performance on the letter cancellation task (100%), and the majority of PCA patients also showed visuospatial (88% at visit 1) and visuoperceptual difficulties (82% at visit 1). In the tAD group, besides impaired performance on visual and verbal memory tasks (as defined by inclusion criteria), patients showed difficulties with visuoperceptual processing (81%) and arithmetic (81%). The PCA group showed a decline in performance on all tests, with a particularly marked drop in performance on the verbal memory task. A decline in performance was also revealed in the tAD group, except on visual memory, letter cancellation, arithmetic, and visuoperceptual processing tests. Formal statistical testing (χ^2^ test) of changes in performance between visit 1 and visit 2 in each group did not reveal significant differences. However, comparing performance at visit 1 between PCA and tAD patients revealed that the PCA group performed significantly better on the verbal memory task (*P* < .001) and arithmetic (*P* = .01), whereas performance in the PCA group was significantly worse on tasks of visuospatial processing (*P* = .01) and the letter cancellation task (*P* = .02) compared with the tAD group.

### 3.2 Boundary shift integral

Table 4 shows the means and standard deviations of the annualized brain atrophy rates in control subjects, PCA patients, and tAD patients, as well as adjusted group differences and *P* values of significance. BSI is reported in milliliters as well as percentage of whole brain volume at baseline. TIV was not significant when included as a covariate in this model (*P* = .3 for milliliter BSI, and *P* = .7 for percentage BSI). Atrophy rates were significantly different between the patient groups and the control group (*P* < .0001 for both PCA and tAD patients) and across all groups (*P* < .0001). The PCA group showed higher atrophy rates compared with the tAD group; however, this difference was not statistically significant (*P* = .08).

### 3.3 Longitudinal gray matter volume changes: control group versus patient groups

**Fig. S1:**
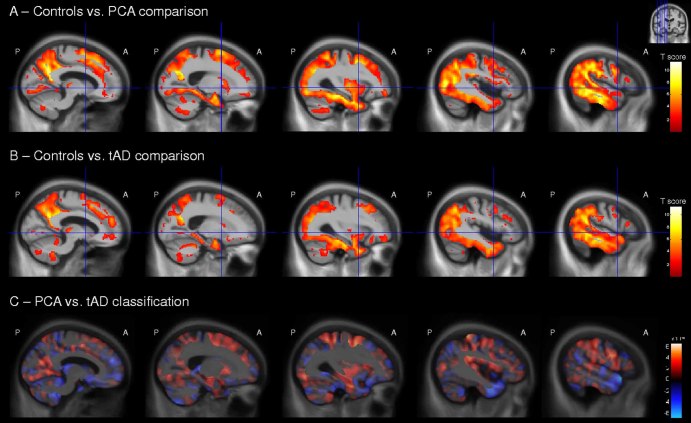
Longitudinal changes in gray matter volume (sagittal view of the left hemisphere) in (A) posterior cortical atrophy (PCA) patients compared with control subjects and (B) typical amnestic Alzheimer’s disease (tAD) patients compared with control subjects. (C) Maps show regions that were most influential in making a classification between PCA and tAD groups. The color bar for the control comparisons (A and B) show *t* values for false discovery rate-corrected results (*P* < .05), with warmer colors indicating greater volume loss in PCA and tAD patients compared with control subjects. (C) Red represents areas where relatively lower gray matter volume change indicates PCA, whereas blue shows areas where lower gray matter volume change indicates tAD. A, anterior; P, posterior.

**Fig. 1:**
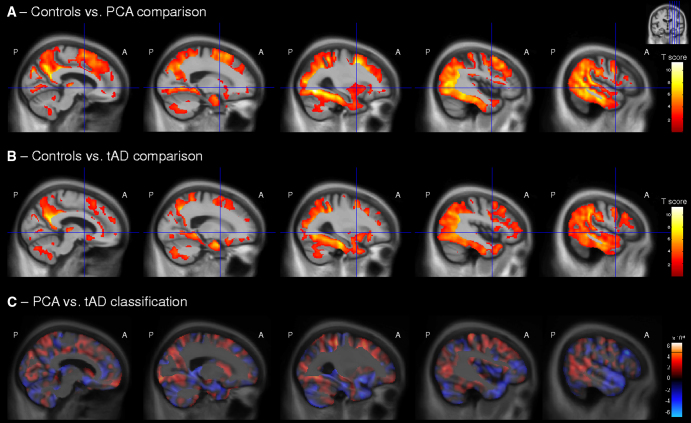
Longitudinal changes in gray matter volume (sagittal view of the right hemisphere) in (A) posterior cortical atrophy (PCA) patients compared with control subjects and (B) typical amnestic Alzheimer’s disease (tAD) patients compared with control subjects. (C) Maps show regions that were most influential in making a classification between PCA and tAD groups. The color bar for the control comparisons (A and B) show *t* values for false discovery rate (FDR)-corrected results (*P* < .05), with warmer colors indicating greater volume loss in PCA and tAD patients compared with control subjects. (C) Red represents areas where relatively lower gray matter volume change indicates PCA, whereas blue shows areas where lower gray matter volume change indicates tAD. A, anterior; P, posterior.

**Fig. 2:**
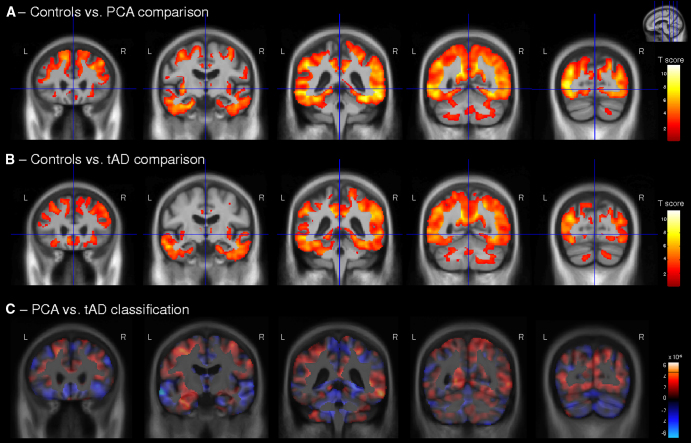
Longitudinal changes in gray matter volume (coronal view) in (A) PCA patients compared with control subjects and (B) tAD patients compared with control subjects. (C) Maps show regions that were most influential in making a classification between PCA and tAD groups. The color bar for the control comparisons (A and B) show *t* values for FDR-corrected results (*P* < .05), with warmer colors indicating greater volume loss in PCA and tAD patients compared with control subjects. (C) Red represents areas where relatively lower gray matter volume change indicates PCA, whereas blue shows areas where lower gray matter volume change indicates tAD. L, left; R, right.

**Fig. 3:**
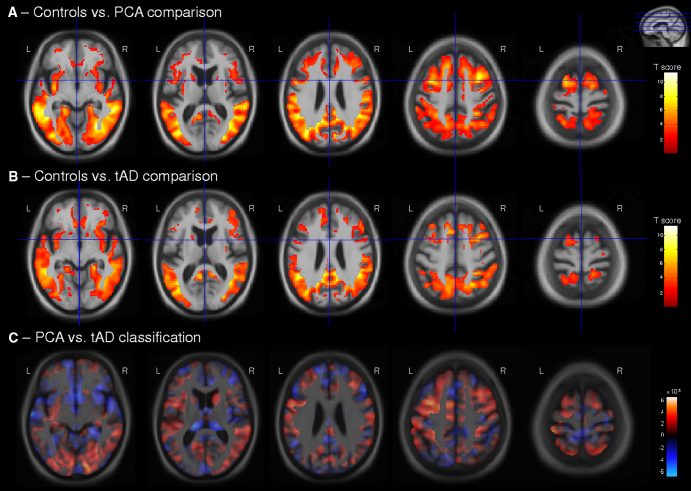
Longitudinal changes in gray matter volume (axial view) in (A) PCA patients compared with control subjects and (B) tAD patients compared with control subjects. (C) Maps show regions that were most influential in making a classification between PCA and tAD groups. The color bar for the control comparisons (A and B) show *t* values for FDR-corrected results (*P* < .05), with warmer colors indicating greater volume loss in PCA and tAD patients compared with control subjects. (C) Red represents areas where relatively lower gray matter volume change indicates PCA, whereas blue shows areas where lower gray matter volume change indicates tAD. L, left; R, right.

Both the PCA and tAD groups showed ongoing global changes in gray matter atrophy compared with the control group (Fig. 1A, B for sagittal view of the right hemisphere, Fig. 2A, B for coronal view, Fig. 3A, B for axial view; please refer to Fig. S1A, B for sagittal view of the left hemisphere). Both groups showed most significant atrophy changes in temporal and parietal lobe regions, with some additional gray matter atrophy changes in the frontal lobe.

### 3.4 Longitudinal gray matter volume changes: PCA patients versus tAD patients

**Fig. S2:**
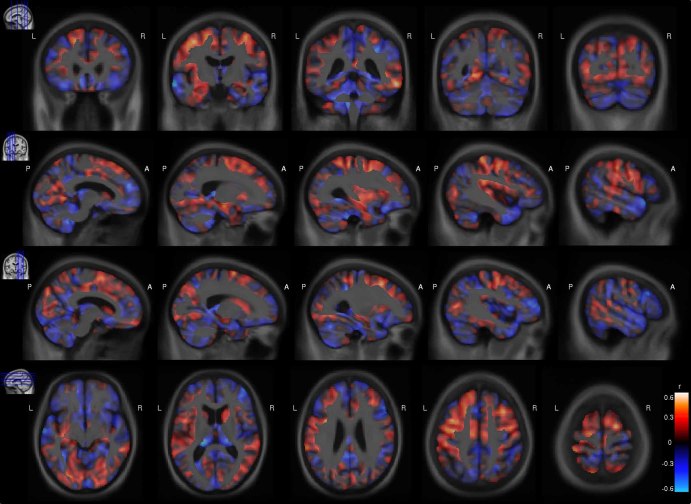
Longitudinal changes in gray matter volume comparing PCA patients with tAD patients. The color bar shows correlation coefficients, with warmer colors indicating greater volume loss in PCA patients compared with tAD patients, and cooler colors representing the reverse contrast. L, left; R, right; A, anterior; P, posterior.

The direct comparison of longitudinal changes in gray matter volume between PCA and tAD groups did not return statistically significant results after FDR correction. Effect size maps (as a Pearson correlation coefficient) for differences in gray matter volume change between these two groups are shown in Fig. S2.

### 3.5 Classification analysis

The results of the classification analysis (accuracies, sensitivities, and specificities) are presented in Table 5. Using the gray matter volume data from the VBM analysis, classification accuracies of 97.1% for PCA patients and 94.1% for tAD patients, against control subjects, were obtained. Sensitivity was 94.1% (specificity 100%) for PCA group and 87.5% (specificity 100%) for tAD group. The direct classification of PCA and tAD groups produced an accuracy of 72.7% (*P* < .01; 95% confidence interval [CI]: 54.5, 86.7), which represents a reasonable classification accuracy, with the confidence intervals indicating that it is significantly different from chance. Specificity for PCA was 75.0% (95% CI: 47.6, 92.7), and sensitivity for tAD was 70.6% (95% CI: 44.0, 89.7).

Regions in which loss of gray matter contributed most to the classification of PCA and tAD groups are presented in Fig. 1C, Fig. 2C, and Fig. 3C. These patterns are very similar to those found in the PCA versus tAD difference map (Fig. S2). The classification map revealed that regions with gray matter loss contributing most to a classification of PCA were the left medial temporal lobe, bilateral supramarginal and inferior parietal lobe, head of the caudate nucleus, and frontal lobe regions. In contrast, regions with atrophy progression contributing most to a classification of tAD were bilateral anterior, middle, and superior temporal lobe regions; frontal lobe regions, including the frontal pole; precuneus; superior parietal lobe; and thalamus.

## 4 Discussion

Patients with AD typically present with amnestic problems and medial temporal lobe atrophy but may less commonly also present with posterior deficits and posterior cortical atrophy (PCA). This study investigated the progression of atrophy in PCA patients compared with tAD patients and healthy control subjects. Compared with control subjects, after a disease duration of 5 years, both PCA and tAD patients showed widespread gray matter loss, including changes in medial temporal and frontal lobe regions in the PCA group and in posterior regions in the tAD group. Differences in atrophy progression between PCA and tAD patients were subtle, reaching statistical significance only when using a multivariate classification approach.

Cross-sectional studies have shown marked focal patterns of atrophy in PCA and tAD patients [14,15], suggesting that at some point during the disease atrophy changes are relatively confined to posterior regions in PCA and to medial temporal lobe regions in tAD. The widespread pattern of gray matter loss in the PCA and tAD groups compared with the control group may therefore suggest that these initial focal patterns of atrophy changes become more global as the diseases progress. This is in accordance with previous reports showing that anterograde memory functions, linguistic skills, and frontal lobe functions, which are sometimes strikingly preserved in the earlier stages of PCA, gradually deteriorate as patients progress to a more global dementia state [10,51], making their presentation ultimately indistinguishable from that found in advanced tAD [52].

The classification analysis produced accuracies of 97% and 94% for discriminating control subjects from PCA and tAD patients, respectively. Accuracy for separating PCA and tAD groups using the same multivariate classification approach was lower (72.7%) but statistically significant. To our knowledge, this is the first study to use a pattern classification algorithm to discriminate between PCA and tAD patients using longitudinal structural MRI data. Previous studies have used classification methods to classify patients with AD, MCI, and frontotemporal dementia, using cross-sectional volumetric and cortical thickness data. Classification accuracies reported for the discrimination between AD and control groups vary between 75% and 100%, depending on measurement unit, classification method, and subject definitions used [53–57]. Accuracies of 84% [54] and 89% [55] have been reported for the discrimination of AD from frontotemporal dementia, and 73% [56] and 82% [58] for the discrimination between MCI-converters versus nonconverters. Wilson et al used an SVM (with principal component dimensionality reduction) to discriminate between different language variants (semantic dementia, progressive nonfluent aphasia, logopenic progressive aphasia) and reported accuracies between 81% and 94% [50]. Lehmann et al reported 98% accuracy for separating PCA patients from control subjects using an SVM and cross-sectional cortical thickness data [59].

Although subject numbers in this study are relatively high, given the rarity of PCA, our study may not have been sufficiently powered to detect statistically significant longitudinal differences between the PCA and tAD groups using the mass-univariate VBM approach. However, the global patterns of atrophy changes in PCA and tAD groups compared with the control group point to a relatively global progression of atrophy in these disease groups. Furthermore, the fact that differences between PCA and tAD patients were sufficient to enable a multivariate classification approach to achieve statistically significant group separation may encourage further studies that assess longitudinal structural changes in larger subject groups. Taken together, our findings support the hypothesis that although regions of greatest total tissue loss in both groups remain those typically associated with these diseases (i.e., posterior regions in PCA, medial temporal lobe regions in tAD), both PCA and tAD patients show global changes in gray matter loss at this stage of the disease.

Whole brain atrophy rates, as measured using the BSI, were approximately 5 times higher in PCA and tAD groups than in the control group. Atrophy rates in the PCA group were slightly higher than in the tAD group; however, the age- and sex-adjusted difference of 0.2%/year was not statistically significant. The mean rate of loss of 1.9%/year found in the tAD group was comparable with losses found in previous studies of approximately 1.6% to 2% per year [17,42,60].

Studies including milder cases of PCA are required to fully understand the evolution of PCA from the earliest symptomatic stages. The recruitment of patients with milder PCA who are closer to symptom onset is often difficult because early visual symptoms are often mistaken as being ophthalmological rather than neurological. Although most baseline images selected for both patient groups (77% for PCA, 75% for tAD) were the first scan available for each subject, the mean disease durations were already 5 years. These durations are also comparable with those reported in other imaging studies of PCA, for example, Mendez et al [34] (PCA: 4.5 years, AD: 4.2 years), Whitwell et al [14] (PCA: 4.0 years, AD: 4.7 years), Schott et al [61] (PCA: 3.8 years, AD: 4.4 years), and McMonagle et al [51] (PCA: 4.5 years). It would also be interesting to study progression of atrophy over multiple time points. Larger group studies will also permit more detailed assessment of the variance within the wider PCA population and, in particular, whether a small number of individuals retain a truly focal pattern of atrophy until late in the disease.

The cognitive and clinical features of the PCA patients included in this study are consistent with those typically reported in PCA studies [7,8,14,34,35]. A large proportion of PCA subjects showed visuospatial and visuoperceptual deficits, as well as dyscalculia, dysgraphia, dyspraxia, and visual disorientation. A number of patients also reported difficulties with their memory as an initial symptom. It should be noted, however, that these subjective memory complaints may not necessarily reflect difficulties with episodic memory per se, but rather may indicate other nonamnestic cognitive symptoms that are difficult for the patient to articulate [62]. This may explain the relatively smaller proportion of individuals with PCA who showed impaired memory performance on formal neuropsychological testing 5 years after symptom onset.

Because the current study aimed to investigate patterns of atrophy changes using MR images, we wished to maximize the number of subjects with comparable image acquisition (i.e., type of scanner and scan interval). For that reason, individuals were drawn from different research studies, which used different neuropsychological assessments. As a result, the psychometric testing data did not permit a more detailed assessment of cognitive progression in these subjects. Further studies that include detailed longitudinal neuropsychological assessments are needed to investigate the progression of cognitive deficits over time in these patients. It would further be interesting to investigate the clinical correlates of the involvement of specific regions in PCA and tAD patients, such as the hippocampus, thalamus, and caudate. In this study, performance on a speed test (letter cancellation), which might represent a neuropsychological correlate for atrophy of subcortical structures such as the thalamus and caudate, was impaired in the majority of PCA patients. However, deficits on this test are also likely to be driven by visual disorientation and visuospatial impairment, potentially making performance on this task less informative in PCA patients. Tests that assess the cognitive processes associated with these subcortical structures and that are less affected by visual dysfunction need to be considered in future studies. Furthermore, because differences in gray matter volume losses between PCA and tAD patients were very small, interpretations of the involvement of these regions should be made with caution.

Although studies have shown that AD is the underlying pathology in the majority of PCA patients [10,11], the syndrome may be attributable to non-AD pathologies in a small number of patients. In this study, pathological confirmation was only available in one PCA patient (AD with additional Lewy body pathology) and four tAD patients (all AD pathology).

In conclusion, this longitudinal study shows that although PCA and tAD patients retain their regional predilection, with PCA continuing to show marked atrophy in posterior regions and tAD in the medial temporal lobe, as the diseases progress, other areas become involved as well, resulting in an increasingly widespread pattern of atrophy progression in both diseases.

## Figures and Tables

**Table 1:** Subject demographics

Characteristics	Control subjects	PCA patients	tAD patients	*P* value∗
N	18	17	16	N/A
Sex, male/female	7/11	6/11	7/9	.9†
Age in years at baseline, mean (SD)	64.0 (5.0)	64.3 (4.7)	72.6 (7.1)‡§¶	<.001
Interval between time points in months, mean (SD)	12.4 (0.6)	12.0 (1.1)	11.6 (0.9)	.06
Disease duration in years at baseline, mean (SD)	N/A	5.1 (2.4)	4.7 (3.1)	.7
MMSE at baseline, mean (SD)	29.4 (0.7)††	21.4 (5.2)‡∗∗	19.3 (3.8)‡∗∗	<.0001
MMSE at follow-up, mean (SD)	29.3 (1.1)††	18.8 (5.9)‡∗∗	16.9 (4.0)‡∗∗	<.0001
TIV at baseline in mL, mean (SD)	1477.9 (131.9)	1466.7 (153.3)	1448.5 (140.1)	.8
Whole brain volume at baseline in mL, mean (SD)	1118.7 (103.1)	983.2 (121.4)‡¶	965.4 (96.2)‡¶	<.0001
Whole brain volume at follow-up in mL, mean (SD)	1120.3 (98.3)	966.1 (129.2)‡¶	949.0 (98.2)‡¶	<.0001

Abbreviations: PCA, posterior cortical atrophy; tAD, typical amnestic Alzheimer’s disease; MMSE, Mini-Mental State Examination; TIV, total intracranial volume.

∗ ANOVA (except sex).

† Fisher exact test.

‡ Compared with control subjects.

§ Compared with PCA patients.

¶ *P* < .001.

∗∗ *P* < .0001.

†† Available for 10 control subjects.

**Table 2:** Frequency of first reported symptoms and clinical features on neurological examination in the PCA group

Symptoms	Percent deficits present
Initial symptoms	
Calculation	71%
Memory	47%
Spelling	41%
Locating objects	41%
Reading	35%
Perceiving distances/depth	35%
Word-finding	35%
Writing	24%
Facial recognition	18%
Identifying objects	12%
Clinical features	
Dyspraxia	47%
Visual disorientation	24%
Myoclonus	6%
Extrapyramidal signs	6%

**Table 3:** Proportion of PCA and tAD patients showing deficits on neuropsychological tests

Cognitive deficits∗	PCA		tAD	
Visit 1†	Visit 2	Visit 1	Visit 2
Memory (verbal)^1^	29%‡	47%	100%	100%
Memory (visual)^1^	–	–	100%	94%
Visuospatial^2^	88%§	94%	44%	81%
Visuoperceptual^3^	82%	88%	81%	81%
Naming^4^	59%	71%	56%	81%
Arithmetic^5^	35%§	41%	81%	75%
Speed^6^	100%¶	100%	69%	63%
Spelling∗∗7	50%	60%	–	–

∗ Proportion (%) of subjects performing below the fifth percentile.

† χ^2^ test comparing baseline assessments between PCA and tAD patients.

‡ *P* ≤ .001.

§ *P* ≤ .01.

¶ *P* ≤ .05.

∗∗ Available in 10 PCA subjects.

1 Short Recognition Memory Test [27] or Easy Recognition Memory Test for words [28].

2 VOSP Number location test [29].

3 VOSP Object Decision or VOSP Silhouettes test [29].

4 Graded Naming test, verbal version of this test in 10 PCA patients [30].

5 Graded Difficulty Arithmetic test [31].

6 Letter cancellation test [32].

7 Graded Difficulty Spelling test [33].

**Table 4:** Overview atrophy rates (BSI) in control subjects, PCA patients, and tAD patients

BSI group characteristics and comparisons	BSI, annualized, in mL	BSI annualized, % of whole brain volume at baseline
Means and SD		
Control subjects	4.0 (4.5)	0.3 (0.4)
PCA patients	22.3 (6.0)	2.3 (0.7)
tAD patients	18.1 (7.4)	1.9 (0.8)
Adjusted differences and confidence intervals		
Control subjects versus PCA patients	18.4 (14.4, 22.3)∗	2.0 (1.6, 2.4)∗
Control subjects versus tAD patients	16.8 (12.0, 21.6)∗	1.8 (1.3, 2.3)∗
PCA patients versus tAD patients	1.6 (−3.2, 6.4)	0.2 (−0.3, 0.7)
*P* (across all groups)	<.0001	<.0001

Abbreviation: BSI, boundary shift integral.

∗ *P* < .0001.

**Table 5:** Classification accuracies, specificities, and sensitivities with 95% confidence intervals in percent for each group comparison for the VBM longitudinal data

Groups	Accuracy	Specificity	Sensitivity
Control subjects versus PCA patients	97.1 (85.1, 99.9)	100.0 (81.5, 100.0)	94.1 (71.3, 99.9)
Control subjects versus tAD patients	94.1 (80.3, 99.3)	100.0 (81.5, 100.0)	87.5 (61.7, 98.5)
PCA patients versus tAD patients	72.7 (54.5, 86.7)	70.6 (44.0, 89.7)	75.0 (47.6, 92.7)

Abbreviation: VBM, voxel-based morphometry.
